# Influenza A Virus Hemagglutinin Antibody Escape Promotes Neuraminidase Antigenic Variation and Drug Resistance

**DOI:** 10.1371/journal.pone.0015190

**Published:** 2011-02-22

**Authors:** Scott E. Hensley, Suman R. Das, James S. Gibbs, Adam L. Bailey, Loren M. Schmidt, Jack R. Bennink, Jonathan W. Yewdell

**Affiliations:** Laboratory of Viral Diseases, National Institute of Allergy and Infectious Diseases, National Institutes of Health, Department of Health and Human Services, Bethesda, Maryland, United States of America; The Scripps Research Institute, United States of America

## Abstract

Drugs inhibiting the influenza A virus (IAV) neuraminidase (NA) are the cornerstone of anti-IAV chemotherapy and prophylaxis in man. Drug-resistant mutations in NA arise frequently in human isolates, limiting the therapeutic application of NA inhibitors. Here, we show that antibody-driven antigenic variation in one domain of the H1 hemagglutinin Sa site leads to compensatory mutations in NA, resulting in NA antigenic variation and acquisition of drug resistance. These findings indicate that influenza A virus resistance to NA inhibitors can potentially arise from antibody driven HA escape, confounding analysis of influenza NA evolution in nature.

## Introduction

The hemagglutinin (HA) and neuraminidase (NA) glycoproteins of influenza A virus (IAV) regulate viral attachment, entry, and release from cells. By binding terminal sialic acids, HA attaches virus to the cell surface to initiate the infectious cycle. By cleaving terminal sialic acids, NA releases virions from cells and substances that can interfere with viral attachment. Maximal viral fitness requires compatibility between the HA and NA, though the underlying mechanisms are incompletely understood.

Oseltamivir and zanamivir are NA active site inhibitors approved for use in humans [1]. Mutants resistant to these drugs are readily selected in cell culture [2], [3], [4], [5], suggesting that widespread use of these drugs has promoted the emergence of drug-resistant IAV strains in humans. This conclusion is supported by the temporal correlation between drug use and emergence of resistant mutants, and more directly by the isolation of NA mutants from chronically infected individuals treated with NA inhibitors [6]. By contrast, epidemiological studies failed to correlate drug use and resistance [7], [8], [9], suggesting the participation of additional factors in selection.

NA-inhibitor resistant mutants are often compensated by changes in HA to restore viral fitness [3], [10], [11], [12]. Many of these changes alter HA affinity for sialic acids. We recently reported that many of the amino substitutions in HA selected for escape from neutralizing monoclonal antibodies (mAbs) modulate HA avidity for host cells [13]. This prompts the question, do some of these substitutions provoke compensatory NA mutations to maximize viral fitness?

## Results

**Figure 1:**
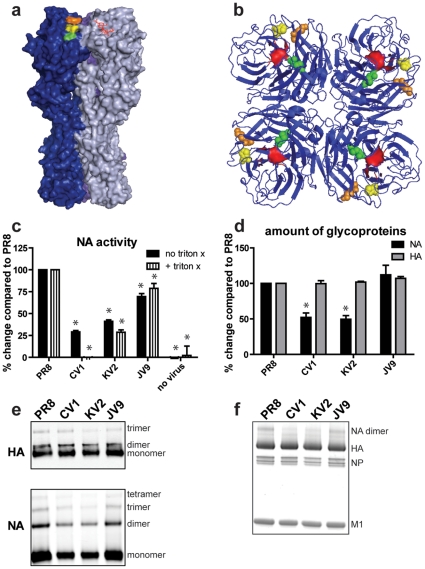
Compensatory NA mutations decrease NA activity. Three-dimensional view of HA trimer (a) and NA tetramer (b). Mutations found in individual viruses are in different colors (yellow = CV1, orange = KV2, green = JV9). Sialic acid (a) and the NA active site (b) are shown in red. (c) NA activities of viruses +/− Triton-X were determined using a colorimetric assay. The amount of glycoproteins incorporated in purified virions was determined by ELISA (d), western blots (e), and total protein blots (f). *, p<0.05 compared to PR8.

We addressed this question by sequencing both HA and NA genes of 40 previously described anti-HA mAb escape mutants (mutants are named based on their selecting mAb). As reported [14], each variant possessed the previously reported mutation leading to an amino acid substitution in their corresponding HA epitopes (Table 1). Three of the 40 viruses, CV1, KV2, JV9, possessed a non-synonymous mutation in their NA genes at positions 274, 253, and 118, while the other 37 viruses had the *wt* sequence. Remarkably, for viruses with NA mutations, the HA substitutions form a clear pattern, since they are all located at the edge of the Sa antigenic site at the HA trimer interface, occupying positions K163T, K165E, and S167F (**Fig. 1a**). Each substitution in NA is located at a different NA residue (**Fig. 1b**).

**Table 1:** Amino acid substitutions in HA mAb-selected variants.

Vrus	HA	NA
BV1	E156G	
BV2	Q196R	
BV6	G159D	
BV11	Q192L	
BV12	N193S	
BV13	N193K	
**CV1**	**K165E**	**H274N**
CV5	P128S	
CV8	N166K	
CV9	P162L	
CV10	L164Q	
DV4	S140P	
DV5	R224I	
DV6	G143R	
EV1	E156K	
EV2	Q192R	
EV8	E198G	
HV1	G173E	
JV3	S167Y	
**JV9**	**S167F**	**V118I**
**KV2**	**K163T**	**K253R**
KV3	N129K	
LV1	R78G	
MV4	L75P	
MV5	S79P	
MV9	V77E	
NV7	S145G	
NV8	S145N	
PV8	S160L	
PV12	N129Y	
PV20	E158V	
RV2	E119G	
RV3	E119K	
RV7	L74P	
SV5	I93T	
SV7	G240R	
WV7	I244T	
WV10	V169A	
WV11	G173R	
WV15	G240E	

The correlation between alterations in this Sa subsite was confirmed by selecting escape mutants with a mixture of Sa-site specific mAbs. Once again, we obtained the K163T substitution, which was accompanied by a T191I NA substitution. Three other escape mutants characterized from the same selection experiment, N129K, N129D, and P162Q had no changes in NA gene sequence. It is nearly impossible statistically for the sequence change in NA that accompanies the K163T mutation to arise from chance in two separate selection experiments performed with different *wt* virus stocks. And it is highly unlikely that chance is responsible for NA sequence changes in the three adjacent Sa residues. Rather, we conclude that amino acid alterations in this region must reduce viral fitness in a manner that selects for compensatory non-synonymous mutations in the NA gene.

What are the effects of these NA substitutions on viral NA activity? CV1, KV2, and JV9 demonstrated reduced NA activity per virion relative to *wt* virus, as measured by hydrolysis of the NA substrate 2′-4-Methylumbelliferyl-alpha-D-N-acetyneuraminic acid (**Fig. 1c**). NA activity was reduced independently of HA physical proximity, as indicated by treating virus with Triton-X 100, a mild detergent that dissociates viral membranes, releasing the glycoproteins from virions. To address the extent to which the mutations reduced NA content per virion vs. intrinsically reduced NA activity, we quantified viral proteins present in purified virions by ELISA (**Fig. 1d**), western blots (**Fig. 1e**), and total protein stain (**Fig. 1f**), normalizing viral quantities by the amount of total protein present in a solution based assay. Reassuringly, the mutant viruses had indistinguishable levels of HA, NP, and M1. Surprisingly, two of the viruses (CV1 and KV2) exhibited reduced amounts of NA relative to other viral proteins. Thus, compensatory NA mutations diminish NA activity by intrinsic effects on enzyme activity and in two cases, by reducing NA incorporation per virion. We did not fully characterize the T191I NA mutation that emerged in our 2^nd^ selection experiments, but based on observation that this amino acid is located far from the NA active site we expect that this mutation likely affects NA activity by reducing NA incorporation into virions, rather than directly altering enzymatic activity.

**Figure 2:**
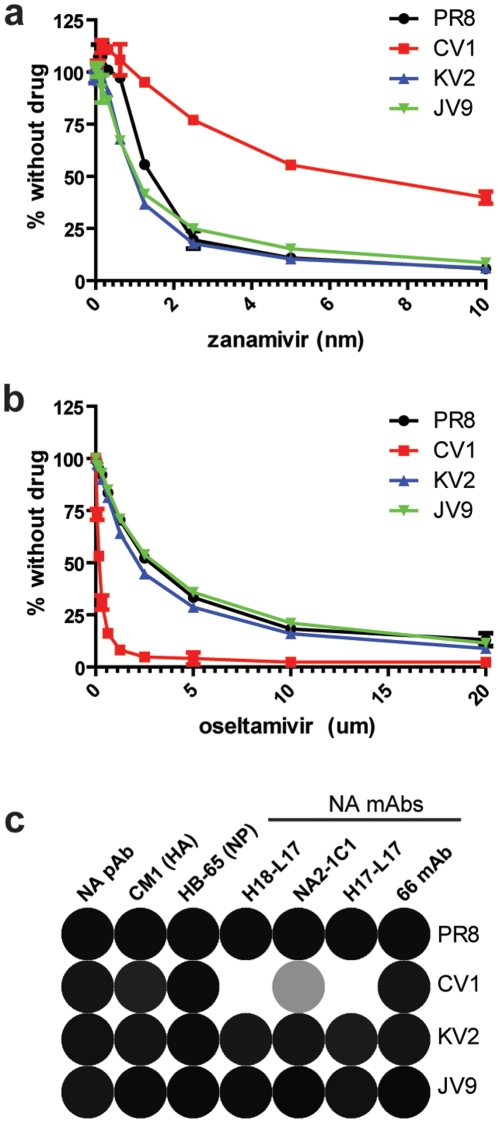
Compensatory mutations alter NA drug sensitivity and antigenicity. Viruses were pre-incubated with zanamivir (a) or oseltamivir (b) and NA activity was determined. (c) Relative binding affinities of 3 control Abs (NA polyclonal Ab, HA mAb, and NP mAb) and 4 anti-NA mAbs were determined using an ELISA assay. Ab titrations were performed and relative K_d_ values were transformed into relative colors using the matrix2png software. Black indicates high binding affinity and white indicates low binding affinity.

NA mutations in CV1 (H274N), and JV9 (V118I) are in close proximity to the active site, while the mutation in KV2 (K253R) is a bit more distant. Substitutions at 253 are known to modulate NA antigenicity [15] and substitutions at residue 274 are known to modulate resistance to oseltamivir [16], [17], [18]. Could the anti-HA mAb escape mutants exhibit altered NA drug sensitivity or antigenicity? We found that CV1 is dramatically resistant to zanamivir and more sensitive to oseltamivir (**Fig. 2a–b**). During the 2008–09 influenza season, the majority of H1N1 IAVs possessed the H274Y NA mutation which promoted oseltamivir resistance. The difference in drug sensitivity between CV1 and the 2008–09 seasonal variants (ie: more sensitive to oseltamivir versus more resistance to oseltamivir) is likely due to the specific amino acid substitution (H274N versus H274Y). Next, we examined NA antigenicity by measuring the binding affinity of anti-NA mAbs to the mutant viruses. Each of the mAbs used is specific for native NA and blocks NA activity using fetuin as a substrate. The mutation in CV1 resulted in a major NA antigenic change, as determined by reduced binding of 3 anti-NA mAbs (**Fig. 2c**). Taken together, these data demonstrate that IAVs can become resistant to NA inhibitors and exhibit altered NA antigenicity in response to Ab pressure on HA, in this particular circumstance, one region of the Sa site in the PR8 HA.

## Discussion

Recent findings indicate that certain mutations in NA allow the molecule to become promiscuous for additional mutations that confer drug resistance [19]. Our findings have similar implications for understanding NA evolution in man: anti-HA Ab driven escape can possibly select for compensatory changes in NA that alter drug resistance. This phenomenon provides a potential explanation for the poor correlation between NA inhibitor use and emergence of drug-resistant strains. Another important implication from our studies is that a significant amount of NA antigenic drift may be a byproduct of HA antigenic drift.

We are uncertain of the underlying mechanism that promotes selection of mutated NA in our experimental system. There are no simple correlations between HA receptor binding avidity and the emergence of compensatory NA mutations. For example, the HA mutation, K165E, increases receptor binding avidity whereas the mutations, K163T or S167F, do not apparently alter receptor binding avidity [13]. It should be noted that these binding assays measure overall avidity to many different glycans expressed on red blood cells, and an intriguing possibility is that NA mutations arise to restore balance to the binding/cleavage to specific glycans. Further elucidation of mechanisms that promote NA compensatory mutations may facilitate early identification of drug resistant IAV strains in the human population.

## Materials and Methods

### Sequencing

RNA was extracted from virus stocks using a QiAmp viral RNA mini kit. For each RNA extraction, we started with 140 ul of virus in allantoic fluid. cDNA was made and HA and NA genes were amplified by PCR. Sequencing was performed on an AppliedBiosystem DNA analyzer.

### Enzyme Activity

NA activity was determined as previously described [20] with slight modifications. For these assays, we used purified viruses. Virus amounts were adjusted based on NA levels as determined by ELISA (for each assay, this corresponded to approximately 50–100 HAUs of virus/sample). Viruses were diluted in assay buffer (33 mM MES (pH 6.5), 4 mM CaCl2) and 200 uM of 2′-4-Methylumbelliferyl-alpha-D-N-acetyneuraminic acid was added. Samples were placed in black flat bottom plates at 37°C and O.D. (Ex = 365 nm; Em = 450 nm) readings were recorded every minute for 30 minutes. Data are expressed as the rate of enzymatic activity (relative vmax). For some experiments, viruses were pre-incubated with 1% Triton-X to disrupt viral membranes. For other experiments, viruses were pre-incubated for 1 hour at 37°C with increasing amounts of oseltamivir or zanamivir.

### ELISAs

For glycoprotein quantification studies, plates were coated with 150 ng/mL of purified virions diluted in PBS (as determined using the BioRad DC protein assay) overnight at 4°C and then blocked for 1 hour at room temperature with PBS-FBS. A saturating amount of either a purified HA mAb (CM1) (diluted 1∶100) or polyclonal rabbit anti-NA Ab mixture (diluted 1∶200) was then added and allowed to incubate for 2 hours at room temperature. Anti-mouse or anti-rabbit HRP (diluted 1∶4000) was then added and allowed to incubate for 1 hour at room temperature. TMB substrate was added and the reaction was stopped with HCL and O.D.s were measured at 450 nm. For mAb escape experiments, plates were coated overnight at 4°C with viruses that were normalized based on NA amount (this corresponds to ∼50–100 HAU/well). The ELISAs were carried out as described above except decreasing amounts of mAb or polyclonal Abs were used. Based on these Ab titrations, K_d_ were calculated and relative binding affinities based on these K_d_ were graphed. For this set of experiments, data were graphed using the previously described matrix2png software [21]. Between all ELISA steps, plates were washed with PBS-tween.

### Immunoblots

Purified virions (3 ug as determined using BioRad DC protein assay) were mixed with SDS-PAGE buffer and run on a 12% SDS-PAGE gel and the gels were blotted on nitrocellulose. These membranes were then incubated with an anti-HA mAb (CM1) (hybridoma supernatant diluted 1∶1) and rabbit polyclonal anti-NA Abs (diluted 1∶1000) and then with infrared dye coupled secondary Abs (Donkey anti-mouse IR Dye 800 and Donkey anti-rabbit IR Dye 680—each diluted 1∶10,000). Blots were analyzed using an Odyssey imaging system. For other experiments, non-reduced samples were run on a 12% SDS-PAGE gel and then stained with Syproruby to visualize total protein content.
